# Psychological Distress and Behavioral Vigilance in Response to Minority Stress and Threat among Members of the Asian American and Pacific Islander Community during the COVID-19 Pandemic

**DOI:** 10.3390/ejihpe14030033

**Published:** 2024-02-26

**Authors:** Andrew S. Franks, Rin Nguyen, Y. Jenny Xiao, Dena M. Abbott

**Affiliations:** 1 Division of Social, Behavioral, and Human Science, University of Washington Tacoma, Tacoma, WA 98407, USA; yxiao2@uw.edu; 2 Department of Counseling Psychology, University of Nebraska Lincoln, Lincoln, NE 68588, USA; knguyen21@huskers.unl.edu (R.N.); dabbott5@unl.edu (D.M.A.)

**Keywords:** anti-Asian prejudice, minority stress, integrated threat, behavioral vigilance, COVID-19, stigmatization

## Abstract

Stigmatization, hostility, and violence towards the Asian American and Pacific Islander (AAPI) community have increased sharply during the COVID-19 pandemic. It is important to conduct research to promote understanding of the effects of such stigmatization on the AAPI community. Accordingly, the present study used a combined minority stress and integrated threat framework to examine whether factors related to AAPI identity would moderate the relationship between stigmatization/threat associated with AAPI identity and increased psychological distress and behavioral vigilance. AAPI individuals were recruited online from both Turk Prime and Reddit and completed measures of perceived stigmatization; integrated threat; depression, anxiety, and stress; and behavioral vigilance. Perceptions of stigmatization and threat predicted relevant outcomes both as individual predictors and in multivariate analyses. However, factors relating to the strength of AAPI identification did not moderate the effects of stigmatization and threat on psychological distress and behavioral vigilance, which is a result that failed to support this aspect of the broader conceptual model on which this project was based. Instead, these proposed moderators were themselves predicted by stigmatization and threat variables. The implications of these findings for effective interventions to alleviate the negative consequences of anti-Asian stigmatization are discussed.

## 1. Introduction

From the Chinese Exclusion Act to the violent coup against the Hawaiian royal family and from Japanese internment camps to the present rise in anti-Asian hate crimes, the Asian American and Pacific Islander (AAPI) community has endured an extensive history of discrimination and violence within the United States (U.S.) [1]. The introduction of the model minority myth [2]—an insidiously dangerous stereotype that cultivates the belief that AAPI people and communities consistently achieve high levels of success compared to other racial and ethnic groups due to their hardworking, submissive, rule-abiding, and resilient nature—brought its own host of damaging effects to the AAPI community and damaged relationships and solidarity between marginalized racial groups [3]. While anti-Asian sentiments have been present in the U.S. since the arrival of Chinese Americans in the 1800s [4], during the COVID-19 pandemic, racist and xenophobic discourse about the origin of the SARS-CoV-2 virus and resulting disease (COVID-19) renewed hostility against AAPI people [5], causing anti-AAPI hate crimes to exponentially increase [6].

Anti-Asian prejudice and stigmatization is a form of minority stress [7]. Minority stress refers to pervasive, socially-based stress associated with a marginalized group membership and, often, the internalization of negative evaluations of the group to which one belongs. Minority stressors, such as the confluence of existing anti-AAPI discrimination and novel and/or resurging forms of prejudice associated with the COVID-19 pandemic, have been shown to predict deleterious mental [7,8,9] and physical health symptoms [10,11]. Scholarship examining minority stress among AAPI people has revealed complex relationships between anti-AAPI discrimination and racism, identification as AAPI or with the AAPI community, and mental health outcomes [12].

The manner in which discrimination, stigmatization, and other forms of racism affect the identity and mental health of AAPI people may be determined by the extent to which AAPI people feel that anti-AAPI racism threatens their symbolic identity (e.g., group beliefs and values) or their lives and well-being. The distinction between these types of threats is the basis for Integrated Threat Theory [13], and, to the best of our knowledge, few or no studies have included integrative threat as a mechanism by which to account for the perception of stigmatization among AAPI people in minority stress models. As higher levels of perceived realistic threats to personal wellness and symbolic threats to group values are associated with psychological distress and behavior changes (e.g., vigilance) [14], it is cogent to build on existing frameworks of AAPI minority stress by evaluating the roles of different types of perceived threat. Accordingly, the current study seeks to examine the degree to which feelings of pandemic-related stigmatization and threat may influence the mental distress and behaviors of AAPI people as well as whether factors relating to racial/ethnic identity may moderate such effects.

### 1.1. COVID-19 Pandemic and Anti-AAPI Racism

The U.S. Department of Justice reported a 77% national increase in anti-Asian incidents from 2019 to 2020 [15]. The most common forms of anti-AAPI racism consisted of verbal harassment (63.0%), physical assault (16.2%), avoidance of AAPI people (16.1%), and civil rights violations (11.5%) [16]. From a different source—one of the largest reporting centers tracking anti-AAPI hate acts in the nation—nearly a hundred daily incidents of anti-AAPI racism were reported within the first week of the COVID-19 pandemic [17], and a total of 11,467 incidents of anti-AAPI discrimination were recorded between the start of the COVID-19 pandemic and the end of March 2022 [18]. As striking as these numbers are, they likely underestimate the actual prevalence of incidents as AAPI individuals are significantly less likely to report experiences of victimization than individuals of other racial and ethnic backgrounds [19]. As a result, many Asian American families have voiced concerns regarding the safety of their children and the potential discrimination and bullying that may persist within their schools and communities [20,21].

Since the start of the pandemic, 177,327 tweets have been made on the social media platform formerly known as Twitter referring to the coronavirus as the “Chinese virus” or the “China virus” [22], and research suggests that social media may be a primary route by which anti-AAPI racism is inflamed [23]. The racialization of the coronavirus from the presidency and the general public [24,25,26] was especially problematic as it cultivated the association between the virus/disease and AAPI communities, and it placed much of the blame for the spread of the coronavirus on AAPI people. These associations and blame further exacerbated xenophobic attitudes and anti-AAPI discriminatory behaviors [27]. Furthermore, anti-Asian beliefs and the use of anti-Asian language were more common in areas with higher levels of support for Donald Trump [28].

These acts of hostility have significantly impacted the economic [29,30], physical [31], and psychological well-being [32,33] of the AAPI community. When examining the economic impact, Asian restaurants welcomed 18.4% fewer customers since the start of the COVID-19 pandemic than non-Asian restaurants, and this pattern was more pronounced in areas with higher levels of support for Donald Trump [34]. When examining psychological impact, the prevalence of depressive symptoms among AAPI individuals more than doubled from 9% before the pandemic to 21% within the first four months of the pandemic [35]. Although the negative impacts of the pandemic have been felt by all groups, a symmetrical increase in levels of mental health disorders has not occurred among the White population, and it is therefore likely that such disparities are at least partially explained by the increased levels of racial hostility faced by the AAPI community during the pandemic [32]. COVID-19-related discrimination was also associated with increased levels of anxiety [12] and post-traumatic stress symptoms (PTS) [36] among AAPI people. Prior research has also shown that experiences of racial discrimination may cause elevated feelings of hypervigilance, avoidance, flashbacks, and nightmares [37], while research conducted in the context of the coronavirus pandemic has shown that pandemic-related psychological distress can lead to dysfunctional coping strategies among other stigmatized groups [38]. Thus, it is likely that AAPI individuals may be experiencing similar negative outcomes within the context of the pandemic.

### 1.2. Minority Stress

Social stress refers to conditions within the social environment that tax an individual and result in negative mental health symptoms when conditions exceed what a person is able to endure [39,40]. Minority stress is a type of social stress occurring as a result of a person’s marginalization and stigmatization relative to dominant groups. Such minority stress is chronic and unique from general and ubiquitous life stressors experienced by all people in that it is a function of the underlying systems of power that create and sustain inequity [7,41]. Originally theorized and applied to individuals of marginalized sexual orientations experiencing heterosexism [7], minority stress has also been applied to people with minoritized racial and ethnic identities experiencing racism and xenophobia [42,43], including AAPI people in the United States [44,45,46,47].

Minority stress is associated with numerous negative mental health outcomes, including mood disorders, anxiety disorders, suicidality, and substance use [7,41]. For example, gay and bisexual men of color with higher levels of minority stress were less able to regulate their emotions compared to those with lower minority stress, they experienced higher levels of depression and anxiety, and, in turn, they reported higher substance use [9]. In a systematic review of the literature, minority stress processes were associated with multiple physiological outcomes including poorer physical health, compromised immune response, and a higher incidence of cancer [11].

Few studies have used minority stress as a framework for studying race-related stressors among AAPI people [43,45,46,48]. More often, studies have examined minority stress-related variables (e.g., race-related stressors and psychological well-being) among AAPI people without using a minority stress framework [49]. Overt forms of racial and ethnic discrimination as well as racial microaggressions (or more subtle and everyday forms of anti-AAPI bias) can increase AAPI people’s level of psychological distress [50]. Among AAPI people, discrimination is significantly associated with negative mental health outcomes [51] such as increased anxiety [52] and depressive symptoms [53,54]. Discrimination was also found to be associated with somatic symptoms [52] and overall poor physical health [53,54]. Racial microaggressions, specifically, are associated with poor mental health outcomes [55,56], poor sleep quality [57], and low well-being [58]. Accordingly, psychological distress is a focal outcome in the current study.

### 1.3. Moderating the Effects of Minority Stress

Cheng et al. [6] proposed a conceptual model that identified a process of collective psychosocial resilience for buffering Asian Americans against the harmful effects of anti-AAPI racism during and beyond the pandemic. The model suggests that the impacts of stigmatization, marginalization, and other contributors to minority stress on AAPI people may be moderated by consciousness-informed racial/ethnic identity, racial solidarity, the dismantling of internalized racism, and advocacy. The current study includes two variables that may function as moderators according to this model: the strength of racial identity and internalized racism.

When one’s marginalized identity is positively associated with social support and coping, it may provide a buffer against the negative impact of racial/ethnic stressors on health [7]. Indeed, for Black college students, a higher commitment to one’s ethnic identity buffered against the deleterious effects of minority stress on higher well-being. Among Latin American adults, ethnic identity commitment operated as a buffer against the stress of daily discrimination [59]. While it is crucial to recognize that feelings of group membership and belongingness may function in unique ways for different groups and identities, the current study’s hypotheses test the prediction that stronger in-group identification will buffer against the effects of stigmatization.

As a function of white supremacy, internalized racism is the acceptance of dominant group superiority (e.g., Whiteness) and the devaluation of the self and others congruent with racism in the dominant society, and it is often associated with negative mental health outcomes [60,61]. Beliefs and attitudes related to internalized racism, such as acceptance of the model minority myth [62], and factors relating to a weakened AAPI identification [63], including racial isolation [64], were some of the main sources of race-related stressors for AAPI people [65]. Specifically, for Asian Americans, the denial or minimization of racism has been theorized to be an important dimension of internalized racism [66]. Importantly, among Asian Americans, the denial or minimization of racism was the only dimension of internalized racism that was consistently associated with lower levels of mental health distress [66]. In the current study, this particular dimension is measured as a potential moderating influence along with the strength of Asian American racial identification. It is important to note that the empirical research conducted by Liao [66] suggests that internalized racism acts as a buffer against the pernicious effects of experiencing and perceiving discrimination, while the model proposed by Cheng and colleagues [6] suggests the opposite. In the current research, both the denial/minimization of anti-Asian American racism and Asian American racial/ethnic identification are hypothesized to buffer against psychological distress and behavioral outcomes, which is consistent with the majority of previous empirical research on the role of such constructs (from both Asian American individuals and other racial groups).

### 1.4. Integrated Threat and the COVID-19 Pandemic

COVID-19 has posed a substantial threat to the health and economic well-being of individuals and groups living in the United States and around the world [67]. The antecedents and consequences of perceiving threat have been studied extensively in the context of intergroup attitudes [68,69]. Specifically, the Integrated Threat Theory conceptualizes perceived threat in two distinct forms—realistic threat and symbolic threat—and it postulates that the two types of threat produce unique outcomes, such as attitudes and behaviors toward social groups [13].

Recently, scholars conceptualized and developed measurements for subjective experiences of both types of threat in the context of the pandemic [14]. In this context, realistic threat concerns the danger of the pandemic to “the physical health and financial well-being of both individuals and their groups”, whereas symbolic threat concerns the pandemic’s effect on “the group’s values and identities, as affirmed by core social processes” [14] (p. 2). Consistent with previous work demonstrating that perceived threat predicts increased distress [70], Kachanoff and colleagues [14] found that both types of threat predicted increased distress and reduced psychological well-being among participants when assessed during the COVID-19 pandemic. In the same study, it was also found that they predicted divergent outcomes in adherence to public health recommendations. Other prior research has shown that perceptions of symbolic and realistic threats within the context of the pandemic may exacerbate racial biases [71]. Based on this research and other evidence from the intergroup threat perspective [70], we predict that perceived realistic and symbolic pandemic threats could predict mental health and behavioral outcomes among AAPI individuals in this research.

### 1.5. The Present Study

As the pandemic and concurrent anti-AAPI racism persist, it is imperative that research examines the impact of pandemic-related hostility and stigmatization on the mental health and daily lives of the AAPI community as well as potential mediating and moderating factors that may buffer against or exacerbate those effects. The current study attempts to achieve this through the lenses of minority stress and integrative threat theories within a recent conceptual model proposed by Cheng and colleagues [6], which posited a multivariate process whereby the effects of broad societal contexts of racism and oppression on adverse outcomes are moderated by factors relating to AAPI identity, solidarity, and advocacy. Based on this conceptual model and the research summarized above, we propose the following pre-registered a priori hypotheses (pre-registered hypotheses as well as data and materials can be found at https://osf.io/q2rsz).

#### 1.5.1. Correlational Hypotheses

**Hypothesis** **1.** *Higher perceptions of anti-Asian stigmatization during the pandemic and higher perceived realistic threat and symbolic threat from anti-Asian stigmatization will predict more severe psychological distress as well as increases in behavioral vigilance*.

**Hypothesis** **2.** *Higher degrees of denying and minimizing anti-Asian racism and stronger Asian American identification will predict lower levels of psychological distress and less behavioral vigilance*.

#### 1.5.2. Regression Hypotheses

**Hypothesis** **3.** *The combination of perceived stigmatization, perceived realistic threat, perceived symbolic threat, denial of racism, and AAPI identification will explain significant variance in both psychological distress and behavioral vigilance*.

**Hypothesis** **4.** *Individual predictors will predict significant unique variance in psychological distress and behavioral vigilance when controlling for each other*.

#### 1.5.3. Moderation Hypothesis

**Hypothesis** **5.** *Denial of racism and group identification will each buffer the effects of stigmatization and threat on psychological distress and behavioral vigilance*.

## 2. Materials and Methods

### 2.1. Participants

Consistent with our pre-registered sampling plan, we attempted to collect participants from three separate samples: workers recruited through Turk Prime, Redditors recruited through AAPI community forums, and students recruited through the psychology subject pool at the first author’s institution. We conducted an a priori power analysis for regression-type models with five predictors, an alpha of 0.05, and a power of 0.80 to detect small effects (*F*^2^ = 0.02) and determined an appropriate total sample size of *N* = 647.

Turk Prime was where we expected to have the most control of the sample size and where we thought our overall sampling efforts would be most successful. We aimed to obtain 447 participants from this pool, and we successfully met this goal while offering USD 1.00 as compensation. Recruitment from Reddit was expected to be much smaller and less controlled, and since Reddit does not have a direct payment system and members of AAPI-focused forums were expected to have intrinsic motivation to participate, these participants were not monetarily compensated. Similar recruitment of Reddit in a previous study [38] resulted in samples of around 150 within a month. After setting 150 as a goal, the final total of Reddit participants in this study was 167 participants. Forty-five participants did not provide enough data to be included in any of the planned analyses, ten of whom indicated at the outset of the study that they were not AAPI and were not invited to continue in the survey.

We initially sought to recruit 50 AAPI psychology undergraduates through the primary author’s university psychology subject pool, but due to low participation (*n* = 5) and the inability to conduct separate analyses on this group, their responses were omitted from analyses. The participants still received course extra credit for completing the study.

The combined sample of Turk Prime and Reddit participants were relatively young (M_age_ = 33.64, SD = 9.79), 53.4% female, and had a median education level of a bachelor’s degree, a median income of between USD 60,000 and 99,999, and a median political orientation of “slightly liberal”. A strong plurality of participants (49.7%) identified as being of East Asian descent, while 29.5% were of Southeast Asian descent, 13% were of South Asian descent, and 9.4% were from the Pacific Islands. Data were collected in April and early May of 2022.

### 2.2. Measures and Procedure

Participants found the study listed either on Turk Prime or in a Reddit forum. Standardized recruitment language accompanied each of the two types of listing (see Appendix A). After completing an informed consent form and confirming their AAPI identity, participants completed the following measures in order. Note that, in all cases, the reported Cronbach’s alpha is higher than the Cronbach’s alpha if any item(s) were removed from any of the scales. Once all measures were complete, Turk Prime participants received a completion code, which they then entered to receive compensation. Reddit users were prompted for feedback and thanked for their time.

Perceived AAPI stigmatization. Perceptions of anti-Asian stigmatization during the pandemic were measured using a series of three items constructed for this study (α = 0.83). An example item is “During the pandemic, I have noticed an increase in anti-Asian hostility on social networking websites”. Participants responded to each item on a scale ranging from 1, “Strongly Disagree”, to 7, “Strongly Agree”. See Appendix B.

Realistic and Symbolic Threat. Perceptions of realistic threat and symbolic threat related to anti-Asian stigmatization were measured on a modified 10-item integrated threat scale [14]. The scale includes two 5-item subscales: realistic threat (α = 0.85) and symbolic threat (α = 0.90). Each item begins with “How much of a threat, if any, is the recent rise in anti-Asian hostility for:” with realistic threat items ending in concerns such as “Your personal health” and symbolic threat items ending in concerns such as “Asian-American traditions and values”. Participants responded on a scale ranging from 1, “Not a threat”, to 4, “Major threat”.

Denial of Racism. The degree to which participants engage in denial or minimization of anti-Asian racism was measured with an 11-item denial/minimization of anti-Asian racism sub-scale from an internalized anti-Asian racism measure (α = 0.91) [66]. An example item is “Racism against Asian-Americans may have been a problem in the past, it is not an important problem today”. Participants responded to each item on a scale ranging from 1, “Strongly Disagree”, to 7, “Strongly Agree”.

Asian American Identification. The strength of participants’ Asian American identification was measured using a modified version (i.e., replacing the generic term “group” with “Asian Americans” as the group of interest) of a 14-item collective identification scale (α = 0.92) [72]. An example item is “I feel solidarity with Asian-Americans”. Participants responded to each item on a scale ranging from 1, “Strongly Disagree”, to 7, “Strongly Agree”.

Psychological Distress. Participants’ psychological distress was assessed using a measure of symptoms of depression, anxiety, and stress, namely the 21-item Depression, Anxiety and Stress Scale (DASS; α = 0.96) [73]. An example item asks whether, during the past week, participants felt that the following statement was true: “I was worried about situations in which I might panic and make a fool of myself”. The scale for each item ranges from 0 to 3, with higher numbers indicating feeling that way more frequently.

Pandemic-Related Behavioral Vigilance. Behavioral vigilance related to perceptions of anti-Asian hostilities during the pandemic was measured using five items developed for this study (α = 0.89). An example item is “Since the start of the pandemic, I have been more alert and vigilant for threats when I have to be out in public”. Participants responded to each item on a scale ranging from 1, “Strongly Disagree”, to 7, “Strongly Agree”. See Appendix C.

### 2.3. Data Analysis

All analyses were conducted using SPSS Version 29 for Windows. After importing data from Qualtrics, composite variables were created using the Compute function. Descriptive statistics were computed using Descriptives and Frequencies functions.

Hypotheses 1 and 2 were tested using two-tailed Pearson’s zero-order correlation analyses with pairwise omission native to the SPSS software. Hypotheses 3 and 4 were tested using linear multiple regression analyses (again, native to the SPSS software) with predictors in all models entered simultaneously. Hypothesis 5 was tested with the external PROCESS macro for SPSS [74] using Model 1 (basic moderation).

## 3. Results

### 3.1. Correlation Analyses

Pearson’s zero-order correlation analyses were conducted between all of the focal variables: perceived stigmatization; realistic threat; symbolic threat; denial of racism; Asian American identification; psychological distress; and pandemic-related behavioral vigilance. Every variable was significantly correlated with every other variable (all *p*s < 0.001) with the exception of Asian American identification and psychological distress (*p* = 0.223). The smallest valid *n* for any bivariate analysis was 574, resulting in an observed power of 1 − *β* > 0.95 for such analyses detecting a small effect (*r* = 0.15) with a conventional Type I error tolerance (α = 0.05).

Consistent with Hypothesis 1, perceived stigmatization of the AAPI community was positively related to the proposed mediators of realistic threat (*r* = 0.522, 95% CI [0.460, 0.579]) and symbolic threat (*r* = 0.576, 95% CI [0.519, 0.628]) as well as to psychological distress (*r* = 0.135, 95% CI [0.054, 0.214]) and behavioral vigilance (*r* = 0.492, 95% CI [0.427, 0.552]). Participants who reported higher perceptions of AAPI stigmatization also tended to report perceiving more realistic and symbolic threats towards the AAPI community and more psychological distress symptoms and behavioral vigilance. In addition, perceived stigmatization was also correlated negatively with denial of racism (*r* = −0.579, 95% CI [−0.630, −0.522]) and positively with Asian American identification (*r* = 0.265, 95% CI [0.188, 0.339]). As participants reported higher perceptions of AAPI stigmatization, they were less likely to deny the existence of anti-AAPI racism—or, to put it more plainly, they were more likely to recognize that anti-AAPI racism exists—and identified more strongly with the AAPI community.

Further, both threat measures were positively related to psychological distress, as measured by the DASS (*r_realistic_* = 0.333, 95% CI [0.259, 0.404]; *r_symbolic_* = 0.247, 95% CI [0.169, 0.322]) and behavioral vigilance (*r_realistic_* = 0.650, 95% CI [0.600, 0.695]; *r_symbolic_* = 0.538, 95% CI [0.477, 0.593]), which was once again consistent with the hypotheses. Participants who perceived greater realistic and symbolic threat tended to report more psychological distress and behavioral vigilance. In addition, both types of threat were negatively correlated with denial of racism (*r_realistic_* = −0.561, 95% CI [−0.615, −0.503]; *r_symbolic_* = −0.609, 95% CI [−0.658, −0.555]) and positively correlated with Asian American identification (*r_realistic_* = 0.309, 95% CI [0.234, 0.381]; *r_symbolic_* = 0.363, 95% CI [0.290, 0.432]). As perceptions of realistic threat and symbolic threat increased, participants were less likely to deny the existence of anti-AAPI racism and identified more strongly with the AAPI community.

Finally, support was mixed for Hypothesis 2 regarding the relationships between the proposed moderators and outcomes. As hypothesized, denial of racism predicted lower psychological distress (*r* = −0.194, 95% CI [−0.271, −0.114]) and behavioral vigilance (*r* = −0.498, 95% CI [−0.557, −0.434]). However, the relationships between Asian American identification and the outcome variables were inconsistent with hypotheses: Asian American identification predicted increased behavioral vigilance (*r* = 0.260, 95% CI [0.182, 0.335]) but it was not significantly related to mental distress (*r* = −0.051, 95% CI [−0.132, 0.031]). Meanwhile, mental distress and behavioral vigilance were, as hypothesized, positively correlated (*r* = 0.257, 95% CI [0.179, 0.332]).

A summary of means, standard deviations, correlation coefficients, and significance values is presented in Table 1.

### 3.2. Regression Analyses

Regression analyses were conducted to determine the combined and unique ability of the proposed predictor variables (including mediators and moderators) to predict outcomes related to psychological distress and behavioral vigilance. The observed power for detecting a very small effect (*F*^2^ = 0.02) at a conventional standard of tolerance for Type I error (α = 0.05) in linear regression models with five and six predictors and 576 (number of participants who filled out the DASS measure) and 574 (number of participants who filled out the behavioral vigilance measures) valid participants, respectively, was 1 − *β* > 0.70. This is below our registered goal of 0.80 given the above parameters, but power for detecting effects as small as *F*^2^ = 0.04 (a medium effect is 0.15) under the above conditions was >0.96. Note that SPSS v29 does not produce confidence intervals for standardized coefficients in a regression analysis.

#### 3.2.1. Predicting Psychological Distress

**Figure 1:**
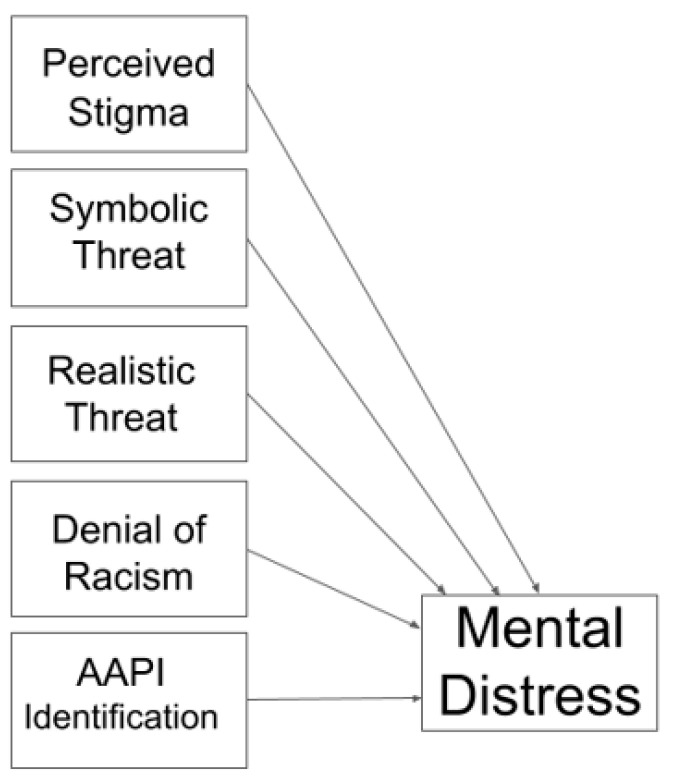
Regression Models Predicting Mental Distress.

Supporting Hypothesis 3, the overall model, including perceived stigmatization of the AAPI community, perceived realistic threat, perceived symbolic threat, denial of anti-AAPI racism, and identification with the AAPI community, predicted a combined significant 14% of the variance in responses on the Depression, Anxiety, and Stress Scale (DASS); *F* (5, 570) = 18.59, *p* < 0.001 (see Figure 1 for model illustration). Among individual predictors, significant unique variance in DASS scores was explained by realistic threat (*β* = 0.326, *p* < 0.001) and by Asian American identification (*β* = −0.119, *p* < 0.001). When controlling for all other predictors, increased perceptions of realistic threat predicted higher psychological distress, while greater Asian American identification predicted lower psychological distress. Both findings were consistent with Hypothesis 4. Other individual predictors did not predict unique variance, but it is probable that some predictors that were significantly correlated with psychological distress at the bivariate level became non-significant in this model due to collinearity with other predictors. Further, Asian American identification became a significant predictor of psychological distress in the multivariate analysis despite being non-significant in a bivariate correlation.

#### 3.2.2. Predicting Pandemic-Related Behavioral Vigilance

**Figure 2:**
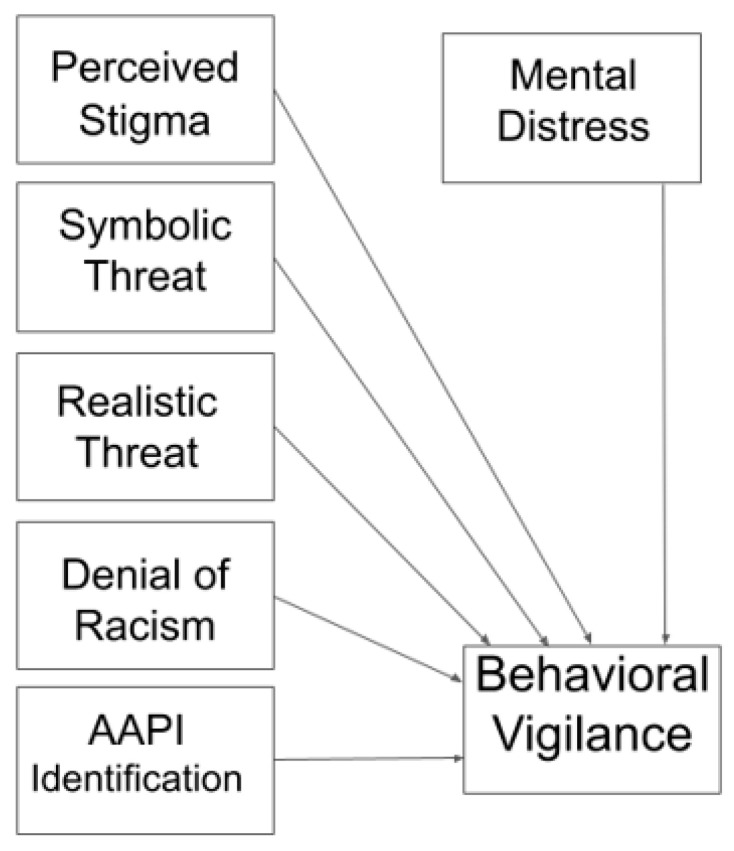
Regression Models Predicting Behavioral Vigilance.

In support of Hypothesis 3, the overall model, including perceived stigmatization of the AAPI community, perceived realistic threat, perceived symbolic threat, denial of anti-AAPI racism, identification with the AAPI community, and psychological distress, predicted a combined significant 47% of the variance in behavioral vigilance (*F* (6, 567) = 83.42, *p* < 0.001) (see Figure 2 for model illustration). Among the individual predictors, perceived stigmatization (*β* = 0.169, *p* < 0.001), realistic threat (*β* = 1.010, *p* < 0.001), and denial of racism (*β* = −0.174, *p* = 0.001), each explained significant unique variance in behavior. When controlling for all other predictors, higher levels of perceived stigmatization and realistic threat each uniquely predicted more behavioral vigilance, while higher levels of denial of racism predicted reduced behavioral vigilance. Each of these findings is consistent with Hypothesis 4. Again, it is probable that other predictors that were significantly correlated with behavior scores at the bivariate level became non-significant in this model due to collinearity with other predictors.

### 3.3. Moderation Analyses

**Figure 3:**
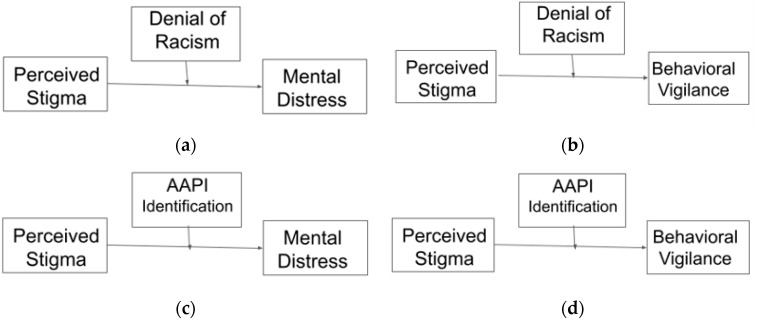
Moderation Models: Predicting Mental Distress (**a**,**c**) and Behavioral Vigilance (**b**,**d**).

In order to test the conceptual model proposed by Cheng and colleagues [6] in which the effects of negative experiences of racial stigmatization on psychological distress and behavioral vigilance are thought to be buffered by stronger in-group identity, we conducted a total of four PROCESS [74] Model 1 analyses testing the ability of denial of racism and Asian American identification to moderate the impact of perceived AAPI stigmatization on psychological distress and behavioral vigilance. All variables were centered. The observed power for detecting a very small effect (*F*^2^ = 0.02) at a conventional standard of tolerance for Type I error (*α* = 0.05) in linear regression-type models with three predictors (x, w, and xw) and 576 and 574 valid participants, respectively, was 1 − *β* > 0.81. The conceptual models are illustrated in Figure 3. Since the unique effects of individual predictors within regression-type models are reported above, in the summary of models below, only the full model and interaction terms are reported as the interaction terms are what is relevant to Hypothesis 5.

#### 3.3.1. Stigma (x), Denial of Racism (w), and Psychological Distress (y)

The combination of stigmatization, denial of racism, and their interaction predicted a significant 4% of the variance in psychological distress, *F* (3, 572) = 8.26, *p* < 0.001. The unique effect of the Stigma × Denial of Racism interaction was non-significant, *b* = −0.0177, 95% CI [−0.043, 0.008], *p* = 0.176.

#### 3.3.2. Stigma (x), Asian American Identification (w), and Psychological Distress (y)

The combination of stigmatization, Asian American identification, and their interaction predicted a significant 3% of the variance in DASS (*F* (3, 572) = 5.18, *p* = 0.002). The unique effect of the stigma × intragroup identification interaction was non-significant (*b* = 0.0035, 95% CI [−0.031, 0.038], *p* = 0.841).

#### 3.3.3. Stigma (x), Denial of Racism (w), and Behavioral Vigilance (y)

The combination of stigmatization, the denial of racism, and their interaction predicted a significant 31% of the variance in behavioral vigilance (*F* (3, 571) = 85.89, *p* < 0.001). The unique effect of the stigma × denial of racism interaction was non-significant (*b* = −0.0122, 95% CI [−0.063, 0.039], *p* = 0.638).

#### 3.3.4. Stigma (x), Asian American Identification (w), and Behavioral Vigilance (y)

The combination of stigmatization, Asian American identification, and their interaction predicted a significant 26% of the variance in behavioral vigilance (*F* (3, 571) = 67.43, *p* < 0.001). The unique effect of the Stigma × Asian American identification interaction was non-significant (*b* = −0.0280, 95% CI [−0.098, 0.042], *p* = 0.433.

### 3.4. A Footnote on Exploratory Analyses

Our OSF registration details plans to conduct exploratory moderated-mediation analyses to examine the ability of threat variables to serve as mediators to any significant moderation relationships. As the moderation analyses above did not show evidence of a buffering effect of either proposed moderator, there was no reason to explore potential mechanisms mediating such interactions.

## 4. Discussion

The present study examined the ability of factors related to the strength of AAPI identity to moderate the relationship between pandemic-related stigmatization/threat and outcomes related to psychological distress and behavioral vigilance among AAPI individuals. Perceptions of stigmatization and threat did indeed predict relevant outcomes both as individual predictors and in multivariate analyses. Denial of anti-AAPI racism also predicted increased psychological distress and behavioral vigilance. However, the denial of anti-AAPI racism and the strength of AAPI identification did not moderate the effects of stigmatization and threat on mental distress and behavioral outcomes, a result that failed to support this particular aspect of the broader conceptual model proposed by Cheng and colleagues [6].

While demonstrating bivariate zero-order relationships is no longer considered necessary recommended practice for conducting multivariate analyses, it was important to report these analyses at the nascent stages of the investigation into the relationships among these variables because if any multivariate hypotheses were unsupported, we may be able to explain such cases by appealing to the bivariate relationships. The patterns of results largely supported the a priori hypotheses. However, in terms of variables proposed to fit into various stages in the moderation models, there were some unforeseen relationships (or lack thereof) regarding the proposed moderating variables. Namely, the proposed moderating variables were found to correlate with what we expected to be the earliest predictor in the causal sequence of our model (perceived stigmatization), meaning that the moderators were less likely than we initially thought to interact with perceived stigmatization in increasing mental distress and vigilance behavior. Further, Asian American identification was not significantly related to psychological distress and was related with behavioral vigilance in the opposite direction than what we hypothesized, making it less likely that Asian American identification (at least as operationally defined for this study) effectively buffers the effect of perceived stigmatization on either outcome. Indeed, this may suggest that the act of reflecting on one’s race or ethnicity and cultural learning may be a stronger predictor of health than simply seeing membership in a racial/ethnic group as an important part of the self [75].

### 4.1. Integration with Previous Scholarship

Our results provide further evidence of the deleterious effects of experiencing such stressors among marginalized groups [7] and, in particular, among AAPI individuals who have recently been enduring increased xenophobic attitudes and discriminatory behaviors [27], and they are consistent with a Minority Stress Framework. While the variables in our model were indeed predictive of psychological distress [55,56], they were even stronger predictors of increased behavioral vigilance. Consistent with the particularly strong relationships between our predictors and behavioral vigilance, our current results demonstrate the importance of accounting for the type and extent of threat perceived by individuals facing the stressors of marginalization and stigmatization in line with Integrated Threat Theory [13]. As mentioned above, the degree to which our results supported Cheng and colleagues’ [6] conceptual model was mixed.

### 4.2. Conclusions

The present results suggest that pandemic-related anti-Asian stigmatization continues to reduce feelings of safety and increase mental distress and vigilance behaviors of AAPI individuals, which reinforces the need for interventions to prevent and buffer against anti-Asian stigmatization, discrimination, and hostility. Attempts at preventing such occurrences may include information and training programs focused on groups and individuals who are likely to perpetrate stigmatization [76], while attempts at buffering the negative consequences of such stigmatization could include training and setting norms for potential allies of the AAPI community to intervene against and confront anti-Asian stigmatization [77] as well as programs meant to promote mental health and feelings of safety among AAPI individuals [78]. Unfortunately, the present study’s attempt to identify specific buffering or mitigating factors was unsuccessful. Rather than moderating the effects of stigma and threat on mental distress and behavioral outcomes, denial of racism and strength of AAPI identification were directly related to measures of stigmatization and threat. This could be because factors identified in the model as moderators affect or are affected by (perhaps as part of additional mediation pathways) the same third variables as the variables they were theorized to moderate. Alternatively, it could be because our specific operationalizations of the proposed moderators did not capture the broader concepts. Regardless, we are unable to make conclusions regarding the causal direction of such relationships.

However, even though racial/ethnic identity did not serve as buffers against perceived anti-AAPI stigmatization, it is fruitful to consider the various alternative theoretical possibilities. One possibility is that racial/ethnic identity-related variables may exist at the first stage of a causal pathway by increasing awareness of stigmatization, which would then subsequently increase threat, psychological distress, and vigilance behavior. The other possibility, and one that would be easier to test, is that feelings of stigmatization and/or threat affect racial/ethnic identity variables. Our research and others point to the importance of studying how racial/ethnic identity may work uniquely for Asian American and Pacific Islanders, and may be different from identity processes and functions for other racial/ethnic groups. While much work has demonstrated a protective effect of a strong racial/ethnic identity against the pernicious mental health consequences of discrimination [79,80], depending on the specific conceptualization and measurement of Asian American racial/ethnic identity, and the specific Asian American sample, some work has found that Asian Americans with a strong ethnic identity actually experienced lower well-being as a result of discrimination [81]. Importantly, the effect sometimes depended on characteristics of utmost importance to understanding the mental health and adjustment of Asian American individuals, namely generational status (e.g., first- vs. second-generation immigration status) [81]. These mixed conceptualizations and research findings, along with our own findings, point to the importance of understanding how racial/ethnic identity functions for Asian Americans compared to other groups, such as Black youths and young adults [75] and Latino adults [59].

### 4.3. Limitations and Future Directions

The conclusions of the current research are limited by the non-experimental methodology. While we are relatively comfortable with concluding that stigmatization and threat are driving changes in depression, anxiety, stress, and vigilance, an experimental study could strengthen such conclusions. In addition, experimental research manipulating experiences of stigma and threat should be conducted to clarify whether denial of racism and AAPI identity are causally related to measures of stigmatization and threat. The current study’s sampling method using online recruitment through MTurk and Reddit likely limited our ability to include older, less tech-savvy individuals and people with low fluency in English. Such individuals may be among the most affected by the processes under examination, and future research should use sampling methods that are more likely to recruit them.

Related to the issue of English fluency is the issue of generational status. We measured and reported this demographic characteristic, but it was not included in any of our models or hypotheses. It may be a critical factor, however, as previous research has indicated that there is an interaction between Asian Americans’ generational status and their strength of ethnic identity in terms of buffering stigmatization’s effects on their mental health [81]. Future research should control for generational status in primary analyses, and we invite other researchers to explore such relationships in our data set.

Yet another limitation is the lack of any buffering effects related to the strength of racial/ethnic identity failing to support our a priori hypothesis based on Cheng and colleagues’ [65] model (Hypothesis 5), which is likely explained by the collinearity of the predictors (including mediators) and proposed moderators reported in the bivariate correlational analyses. As such, the proposed moderators may have played a different role than what we hypothesized. For instance, racial/ethnic identity-related variables may have caused individuals to be more likely to be aware of stigmatization, which would then subsequently elevate threat, mental distress, and vigilance behavior. Future research should explore additional possible moderating factors to serve as interventions to prevent experiences of stigmatization and threat or to buffer their effects.

In addition, future research may seek to examine the symmetry or asymmetry of bias between Asians as a marginalized group and Whites as a majority group [82], stigma by association [83] among the non-Asian friends and allies of AAPI people, and the role of politically motivated cognitions on memory [84] of stigmatizing events among AAPI people.

Finally, this study examined outcomes related to mental health in a general population sample of Asian Americans and found that stigmatization, realistic threat, and symbolic threat predicted behavioral vigilance more strongly than they predicted anxiety and depression symptoms. It is possible that there are stronger relationships between stigmatization and threat and mental health outcomes in a clinical sample suffering from clinical levels of anxiety and/or depression, and further research may sample from individuals in clinical populations.

## Figures and Tables

**Table 1:** Means, standard deviations, and Pearson’s zero-order correlations (below diagonal) and *p*-values (above diagonal).

Variable	M	SD	1	2	3	4	5	6	7
1. Perceived Stigma	5.28	1.43	-	<0.001	<0.001	<0.001	<0.001	<0.001	<0.001
2. Realistic Threat	2.41	0.73	0.522	-	<0.001	<0.001	<0.001	<0.001	<0.001
3. Symbolic Threat	2.68	0.84	0.576	0.614	-	<0.001	<0.001	<0.001	<0.001
4. Denial of Racism	14.75	1.17	−0.579	−0.561	−0.609	-	<0.001	<0.001	<0.001
5. Asian American ID	17.35	0.95	0.265	0.309	0.363	−0.350	-	0.223	<0.001
6. Psych Distress	1.76	0.65	0.135	0.333	0.247	−0.194	−0.051	-	<0.001
7. Behavioral Vigilance	16.15	1.53	0.492	0.650	0.538	−0.498	0.260	0.257	-

## Data Availability

The study data are available at https://osf.io/q2rsz.

## Appendix A. Recruitment Language

This study is being conducted by faculty in the Division of Social, Behavioral, and Human Sciences at University of Washington Tacoma. You are being recruited as a member of the Asian/Asian Americans and Pacific Islander (AAPI) community in the United States. You must be over 18 years old and unaffiliated with the University of Washington to participate.

If you choose to participate, you will be directed to an online survey to answer a host of surveys that measure aspects of yourself and your experiences during the COVID-19 pandemic. The study will take place in one session and will take about 10 minutes. You are being recruited because you may be particularly impacted by the phenomena we are studying. This study is completely confidential. We do not collect any identifying information, so your answers will not be linked to your name or any other identifiable information.

## Appendix B. Perceived AAPI Stigmatization

Each item was measured on a 1 (Strongly Disagree) to 7 (Strongly Agree) scale.

1. During the pandemic, I have noticed more negative depictions of Asian-Americans in the media.
2. During the pandemic, I have noticed an increase in anti-Asian sentiment expressed by political leaders.
3. During the pandemic, I have noticed an increase in anti-Asian hostility on social networking websites.

## Appendix C. Pandemic-Related Behavioral Vigilance

Each item was measured on a 1 (Strongly disagree) to 7 (Strongly agree) scale.

1. Since the start of the pandemic, I have been doing more to actively avoid anti-Asian harassment.
2. Since the start of the pandemic, I have been leaving the house less often due to a fear of being harassed for being Asian-American
3. Since the start of the pandemic, I have been more alert and vigilant for threats when I have to be out in public.
